# Comparing Species Composition of Passive Trapping of Adult Flies with Larval Collections from the Body during Scene-Based Medicolegal Death Investigations

**DOI:** 10.3390/insects8020036

**Published:** 2017-03-24

**Authors:** Michelle R. Sanford

**Affiliations:** Investigations Division, Harris County Institute of Forensic Sciences, 1861 Old Spanish Trail, Houston, TX 77054, USA; michelle.sanford@ifs.hctx.net; Tel.: +1-832-927-5048

**Keywords:** Diptera, colonization, casework, decomposition

## Abstract

Collection of insects at the scene is one of the most important aspects of forensic entomology and proper collection is one of the biggest challenges for any investigator. Adult flies are highly mobile and ubiquitous at scenes, yet their link to the body and the time of colonization (TOC) and post-mortem interval (PMI) estimates is not well established. Collection of adults is widely recommended for casework but has yet to be rigorously evaluated during medicolegal death investigations for its value to the investigation. In this study, sticky card traps and immature collections were compared for 22 cases investigated by the Harris County Institute of Forensic Sciences, Houston, TX, USA. Cases included all manner of death classifications and a range of decomposition stages from indoor and outdoor scenes. Overall, the two methods successfully collected at least one species in common only 65% of the time, with at least one species unique to one of the methods 95% of the time. These results suggest that rearing of immature specimens collected from the body should be emphasized during training to ensure specimens directly associated with the colonization of the body can be identified using adult stages if necessary.

## 1. Introduction

Insects can provide useful data in death investigations. While they have the potential to provide many types of information, the most widespread application of forensic entomology data in death investigations is the estimation of the time of colonization (TOC) by insects [1]. The estimation of the TOC is based on the assumption that insect colonization occurs after death and that insects have the potential to colonize a body shortly after death, and therefore the TOC may approximate the time since death or the post-mortem interval (PMI) [2,3,4].

Using accumulated degree days (ADD) or accumulated degree hours (ADH) is one method employed by forensic entomologists to estimate the TOC [5]. This method relies on the property of insects to be poikilothermic, thus requiring external heat for a majority of their developmental needs. Via knowledge of a particular species’ temperature development thresholds and observations of developmental milestones in the laboratory, the heat units required for each stage can be determined. In application, the ADH method requires insect samples of the oldest life stage of the primary colonizing insect species on the body, published laboratory development data obtained from populations with a geographically similar history and a climatically similar laboratory temperature regime for the species collected from the body, as well as an accurate temperature history for the location of the body to generate informative and reliable estimates of TOC [5,6,7,8,9]. The use of the ADD or ADH method [5] has been widely adopted for forensic entomology casework.

An ADH method–based case uses a targeted approach to sampling focused on obtaining the oldest species and the most developed life stages present on and around the body as they presumably represent the primary colonizers, which in turn provide the most accurate indicators of PMI. Succession-based PMI estimation, a widely researched ecologically based technique for PMI estimation [2], is more focused on ascertaining the entire community at the time of body recovery and is therefore focused on sampling as many insect specimens as possible. Numerous publications have detailed instructions related to insect sampling at both scenes and autopsies [3,10,11,12], but this dichotomy in TOC/PMI estimation as reflected in sampling has not been formally addressed. These methods typically focus on the collection of large numbers of insects of all life stages, from all areas of the body and scene. However, the relationship between adult flies and decedents at scenes during the course of medicolegal death investigations may not always indicate colonization relevant to the PMI.

Indoor scenes comprise approximately two-thirds of the forensic entomology cases, represented by all manners of death, analyzed by the Harris County Institute of Forensic Sciences (HCIFS) [13]. Scene investigation comprising the many required law enforcement and medicolegal death investigation procedures may take time. Once a previously sealed indoor location is entered, from the discovery of the decedent and crime scene processing through the recovery of the body, insects and particularly flies have newly gained access. At outdoor scenes the movement and manipulation of the body during the investigation can also attract flies from the environment as the odors at the scene change. These flies have no informative value to the PMI estimate given that they arrived after the decedent was known to be deceased and might be considered contaminants to the scene. Egg deposition can even occur due to these new fly arrivals during scene investigation [14].

There are two widely adopted methods for collecting adult flies on the scene including: active sampling with a sweep net and passive sampling with a sticky trap [3,11]. The sticky trap approach has advantages at indoor scenes where it is impractical to use a sweep net and in investigations that require trace and DNA evidence recovery, due to the potential for contamination issues associated with swinging a net over the body. With a practical approach to ADH-based TOC estimation in casework in mind, the simple question was asked: do the flies collected with a sticky trap at indoor and outdoor death investigation scenes match the identity of the fly immatures collected from the body? This question was approached by placing sticky card traps during routine scene death investigations involving the collection of entomology samples (consisting primarily of larval fly samples), handled by the HCIFS for 22 cases.

## 2. Materials and Methods

**Cases**—The cases included in this analysis consist of 22 scene deaths investigated by the Harris County Institute of Forensic Sciences (HCIFS) where the forensic entomologist attended the scene and collected larval flies from the decedent’s body and adult flies from the scene with the use of a passive sticky trap. All of the samples from these cases were used to calculate an estimated TOC using the ADH method [5] as part of the forensic entomology analysis conducted on each case. Table 1 provides details regarding the time of year, location of the body and official manner of death for the cases analyzed.

In addition to the comparison of species composition on trapping method, the presence of male and female blow flies collected on the sticky trap was recorded for comparison to data collected by Mohr and Tomberlin [15]. However the stage of decomposition for the cases described here differs from that observed by Mohr and Tomberlin [15]. The HCIFS uses a five stage broad classification system for the stages of decomposition, including no decomposition, early (skin slippage, marbling), moderate (bloating, conspicuous insect activity), advanced (bone exposed) and skeletal. These decomposition stages have been related to previously published literature [16]. The cases described here range from the early stages to the advanced stage.

**Collection Methods**—Larval fly specimens were collected from the body according to HCIFS standard operating procedures which are based on several sources [2,3,4,12]. This includes collection of multiple samples from foci of larval fly activity on the body including, natural body openings, wounds and other areas of activity depending on the circumstances of each case. Preservation of a portion of the specimens was accomplished by hot water kill and placement in 70% ethanol [12,17] and the remainder of the specimens were reared to confirm identification. Identification of the larvae was accomplished with the use of taxon and life stage appropriate keys (e.g., [18,19,20,21,22]).

A single sticky trap (Catchmaster®, Mouse and Insect Glue Traps, Heavy Duty, Pre-baited, AP&G Co., Inc., Brooklyn, NY, USA) was placed within approximately 30 cm of the body at each scene following the method of Haskell and Williams [23] in a “pup tent” configuration but without the clothes pins on the bottom of the trap. Traps were deployed for 15 minutes to one hour during investigation. Following deployment, the traps were folded over, placed into an evidence bag and placed into a standard freezer for preservation and storage prior to identification. The adult fly specimens were identified with use of appropriate keys and descriptions (e.g., [24,25,26]).

## 3. Results and Discussion

The 22 cases reviewed in this study revealed a lack of congruence between the fly specimens collected via the passive sticky trap and those collected as immatures from the body during death scene investigations. Only 65% of the cases had at least one species that was the same using both methods. The cases included both indoor and outdoor scenes; however, a majority were indoor scenes. The manner of death for the cases included several different manners (Table 1); however, no overall trend was observed that might explain the discrepancy in collection methods. One point often raised with regard to collection of adult fly samples at the scene is that they help to confirm the species identity of immature specimens collected from the body. However, these data suggest that their utility, as collected by sticky trap, does not bear this out. Importantly, this study was limited by sample size and continued sampling may help to elucidate the types of scenes where the collection of adults may prove useful. The challenge that will pose a problem for repeated sampling will be the almost infinite number of scenarios that one encounters during medicolegal death investigation scenes.

Species collected differed for both indoor and outdoor scenes, for the observed stages of decomposition, and months and manners of death. There is one exception in these data, however: all three of the cases with the official manner of death of accident were accidental overdoses related to acute cocaine toxicity (one coupled with chronic ethanol abuse). In these three cases, both of these collection methods yielded the same species. Two of these accidental cases were located indoors and one was located outdoors and all three were moderately decomposed (Table 1). Cocaine is known to have effects on the larval development and size of some fly species [27] and other drugs with similar properties also have effects on development and size [28], but the effects on fly attraction and colonization are unknown.

Overall more female flies (115) were collected than males (33). This would appear to be consistent with changes at the scene either allowing additional opportunities for colonization or in changing the attractive nature of the scene to additional flies. However, there did not appear to be a trend associated with the variables recorded in this limited study.

An often-overlooked aspect of scene investigation is the impact of the investigation itself on insect access. Insects can gain access to the scene once the scene is entered by first responders and there can be hours between the arrival of first responders and the forensic entomologist or medicolegal death investigator who may collect specimens [14]. This complicates the matter further because it then becomes impossible to determine which flies were associated with the body before the body was accessed, which arrived when access was made and which are just arriving. Furthermore, if gravid females can be used in some way to generate a colonization estimate [29], there is a possibility that the newly acquired access that has been made to the body will alter the TOC estimate made with this method. This new access can also complicate the use of blow fly eggs in TOC estimation, which can be deposited during investigation [14]. These observations support the use of larval and pupal insects that have an established colonization time of the body for more accurate TOC estimation whenever they are available. Adult flies that are dead or that are newly emergent can be collected by other means [12] and are much more likely to be directly associated with the body and potentially useful for the TOC estimate. Taken together, these data suggest training collection staff to obtain samples for rearing as part of standard procedures. Furthermore, they underline the importance of context appropriate validation efforts in forensic entomology.

## Figures and Tables

**Table 1:** Month, location of the body at the scene, manner of death, stage of decomposition and species collected via sticky trap or as immatures reared from the body for fly specimens collected from 22 cases investigated by the Harris County Institute of Forensic Sciences from 2013–2014. The numbers of male and female blow flies collected are also listed for each sticky trap collection.

Case #	Month	Scene Location	Manner of Death	Decomposition Stage	Species on Trap	M	F	Species on Body
1	May	Outdoor	Undetermined	Advanced	*Lucilia cuprina*	0	1	*Chrysomya rufifacies*
					*Musca domestica*			*Lucilia cuprina*
					Piophilidae			*Lucilia coeruleiviridis*
2	June	Outdoor	Homicide	Moderate	*Chrysomya megacephala*	16	6	*Cochliomyia macellaria*
					*Chrysomya rufifacies*	3	9	*Chrysomya rufifacies*
					*Lucilia eximia*	0	4	
					Sarcophagidae			
					Muscidae			
					Piophilidae			
					Sciaridae			
3	July	Outdoor	Homicide	Moderate	No flies collected			*Cochliomyia macellaria*
		(car)						*Chrysomya rufifacies*
								*Chrysomya megacephala*
4	August	Outdoor	Natural	Moderate	*Chrysomya megacephala*	1	4	*Chrysomya megacephala*
					*Chrysomya rufifacies*	1	4	*Chrysomya rufifacies*
5	September	Outdoor	Accident	Moderate	*Chrysomya rufifacies*	1	0	*Chrysomya rufifacies*
					Piophilidae			
6	May	Indoor	Natural	Moderate	*Lucilia cuprina*	0	1	*Lucilia cuprina*
								Sarcophagidae
								Phoridae
7	June	Indoor	Natural	Moderate	*Cochliomyia macellaria*	5	22	*Cochliomyia macellaria*
					*Chrysomya megacephala*	2	17	*Chrysomya megacephala*
					*Chrysomya rufifacies*	0	6	Sarcophagidae
					*Lucilia sericata*	0	1	Phoridae
					*Lucilia cuprina*	0	1	
					Phoridae			
8	July	Indoor	Natural	Moderate	*Chrysomya megacephala*	4	7	Calliphoridae
					*Cochliomyia macellaria*	0	3	
					*Chrysomya rufifacies*	0	2	
9	August	Indoor	Natural	Moderate	*Chrysomya rufifacies*	0	2	Sarcophagidae
					Sepsidae			
10	September	Indoor	Suicide	Early	No flies collected			Diptera (fly eggs)
11	September	Indoor	Natural	Early	No flies collected			*Blaesoxipha plinthopyga*
								*Chrysomya rufifacies*
								*Chrysomya megacephala*
								Muscidae
12	October	Indoor	Accident	Moderate	*Chrysomya rufifacies*	0	1	*Chrysomya rufifacies*
					*Chrysomya megacephala*	0	1	*Chrysomya megacephala*
					*Lucilia cuprina*	0	1	*Blaesoxipha plinthopyga*
					*Hydrotea* sp.			
13	October	Indoor	Accident	Moderate	*Chrysomya rufifacies*	0	2	Sarcophagidae
					*Chrysomya megacephala*	0	2	*Chrysomya rufifacies*
					*Megaselia scalaris*			*Megaselia scalaris*
14	October	Indoor	Natural	Moderate	No flies collected			Sarcophagidae
								*Chrysomya megacephala*
								Phoridae
15	October	Indoor	Natural	Advanced	*Megaselia scalaris*			Sarcophagidae
					Sarcophagidae			*Chrysomya megacephala*
								*Megaselia scalaris*
16	November	Indoor	Undetermined	Moderate	*Chrysomya megacephala*	0	1	*Blaesoxipha plinthopyga*
					Sarcophagidae			
17	January	Indoor	Natural	Early/Moderate	*Chrysomya megacephala*	0	1	Phoridae
					Drosophilidae			*Megaselia scalaris*
								
18	June	Indoor	Natural	Moderate	*Cochliomyia macellaria*	0	6	*Phormia regina*
					*Lucilia cuprina*	0	1	Phoridae
					*Megaselia scalaris*			
					Spaeroceridae			
					*Hydrotea* sp.			
19	June	Outdoor	Homicide	Moderate	*Chrysomya rufifacies*	0	1	*Cochliomyia macellaria*
					*Cochliomyia macellaria*	0	1	*Chrysomya rufifacies*
					*Lucilia cuprina*	0	1	*Chrysomya megacephala*
					*Musca domestica*			*Lucilia spp.*
								*Phormia regina*
20	July	Outdoor	Natural	Moderate	*Chrysomya rufifacies*	2	20	*Chrysomya rufifacies*
					*Chrysomya megacephala*	1	12	*Lucilia eximia*
					*Lucilia cuprina*	0	7	*Chrysomya megacephala*
					*Muscidae*			
21	July	Outdoor	Homicide	Moderate	Muscidae			*Lucilia eximia*
								*Chrysomya rufifacies*
								*Cochliomyia macellaria*
22	September	Outdoor	Natural	Moderate	*Cochliomyia macellaria*	6	17	*Chrysomya rufifacies*
					*Chrysomya megacephala*	6	23	*Cochliomyia macellaria*
					*Chrysomya rufifacies*	4	41	*Lucilia* sp.
					*Lucilia cuprina*	0	1	*Chrysomya megacephala*
					Muscidae			
